# Bundle Branch Blocks and Fragmented QRS Complex in Iranian Patients with Systemic Sclerosis

**Published:** 2019-01

**Authors:** Ali Javinani, Zahra Javady Nejad, Farhad Gharibdoost, Ahmad Reza Jamshidi, Reza Atef Yekta, Saba Alvand, Vahide Imeni, Seyed Naser Hashemi, Hoda Kavosi

**Affiliations:** 1 *Rheumatology Research Center, Tehran University of Medical Sciences, Tehran, Iran.*; 2 *Department of Anesthesiology, Shariati Hospital, Tehran University of Medical Sciences, Tehran, Iran. *

**Keywords:** *Sclerosis*, *Electrocardiography*, *Bundle-branch block*

## Abstract

**Background:** Cardiac involvement, as one of the life-threatening manifestations of systemic sclerosis (SSc), is chiefly caused by collagen fiber deposition in the myocardium, which subsequently leads to conduction abnormalities. In the present study, we aimed to investigate the prevalence and clinical significance of bundle branch blocks (BBBs) and the fragmented QRS complex (fQRS) in Iranian patients with SSc.

**Methods:** Forty-one patients with SSc were enrolled from the outpatient SSc clinic of Shariati Hospital, Tehran University of Medical Sciences, between October 2016 and February 2017. Twelve-lead ECG was obtained and interpreted for BBBs and the fQRS. To adjust for the confounding effects of non–SSc-related cardiovascular risk factors, we calculated the Framingham risk score to estimate the risk of cardiovascular diseases. The associations between the studied conduction abnormalities and SSc cutaneous subtypes; disease duration; and the Medsger SSc severity scale of cutaneous, pulmonary, and vascular involvements were also analyzed.

**Results:** The study population consisted of 41 Iranian patients with SSc at a mean age of 47.48±11.57 years (82.9% female). The prevalence of BBBs and the fQRS was 26.8% and 36.6%, respectively. The fQRS was associated with the limited cutaneous SSc subtype (OR: 0.100, 95%CI: 0.018–0.553, and P=0.028). BBBs and the fQRS were not associated with either the Framingham risk score or the rest of the clinicodemographic variables.

**Conclusion:** BBBs and the fQRS were more prevalent in our patients with SSc, without any association with the involvement of the other organs. These findings may suggest the independent pathophysiology of cardiac involvement in SSc.

## Introduction

Systemic sclerosis (SSc) is an autoimmune disease with a multi-organ involvement. Cardiac abnormalities are life-threatening manifestations which can be present in all parts of the heart structures. Cardiac conduction defects are one of these abnormalities, with an approximate prevalence of 25% to 75%.^        1 ^ According to the European League Against Rheumatism (EULAR) Scleroderma Trials and Research (EUSTAR) database, conduction defects account for 6% of patient mortality.^        2 ^ In addition, the report of the Genetics Versus Environment In Scleroderma Outcome Study (GENISOS) cohort showed that cardiac arrhythmias were an independent predictor of mortality.^        3 ^

The presence of ectopic and excessive collagen fibers in the myocardium and the bundle branches is the most probable cause of these conduction defects.^        1 ^ Similarly, the new phenomenon of the fragmented QRS complex (fQRS) may be linked to this pathologic pathway.^        4 ^ The fQRS is an indicator of conduction defects which has recently entered the field of cardiology; it was initially proposed as a marker in identifying the previous myocardial ischemic changes similar to the Q wave. Several lines of evidence, thus, suggest that the presence of the fQRS is an important prognostic factor in patients with the acute coronary syndrome and hypertension.^5^^-^^7^

There are several methods for detecting cardiac involvement, but the electrocardiogram (ECG) is still the most common way for detecting defects of the conduction system, partly because of its availability and cost-effectiveness. ECG can also expose variable dysrhythmias in asymptomatic patients.

Little research has been devoted to addressing the clinical significance of conduction abnormalities in SSc, but it appears that it is a consequence of myocardial fibrosis. Most of the evidence suggests that the fQRS is more prevalent among patients with SSc; nonetheless, its clinical significance is not fully elucidated and findings from different studies are highly incongruent.^   8 ^ In this study, we collected normal 12-lead ECGs from 41 Iranian patients with SSc with a view to specifically assess bundle branch blocks (BBBs) and the fQRS and their association with demographic and clinical variables in patients suffering from SSc. 

## Methods

Forty-one Iranian patients who fulfilled the 2013 classification criteria for SSc, an American College of Rheumatology (ACR)/EULAR collaborative initiative, were enrolled in the study from the outpatient SSc clinic of Shariati Hospital, Tehran University of Medical Sciences, via the simple random sampling approach.^        9 ^ Data collection was begun in October 2016 and was finished in February 2017. During that period of sampling, the fixed sampling framework consisted of 100 patients suffering from SSc with a female-to-male ratio of approximately 5 to 1. The subtypes of SSc were determined according to the ACR classification, and disease duration was calculated from the appearance of the Raynaud phenomenon to the time of the study.^        9^

Cutaneous, pulmonary, and vascular involvements were considered for the analysis by using the Medsger SSc severity scale (MSSS).^   10 ^ The MSSS is a 0-to-4 scale defined separately for each organ to measure the severity of its involvement. The skin MSSS is determined according to the extent of skin fibrosis measured using the modified Rodnan skin score (mRSS).^   11 ^ This index is a 0-3 scoring system of 17 areas and is used to evaluate the skin stiffness of the patient. The mRSS was calculated by a single rheumatologist with an acceptable reliability coefficient over time (ICC=0.88). The lung MSSS is calculated by the combination of the 3 variables of pulmonary arterial pressure (PAP), forced vital capacity, and diffusion capacity for carbon monoxide. A single instrument was used to obtain forced vital capacity and diffusion capacity for carbon monoxide, and the PAP was estimated by echocardiography. The vascular MSSS is also defined by the severity of peripheral vascular involvement, including the presence of the Raynaud phenomenon or pitting digital ulcers. 

Twelve-lead ECGs were obtained at the time of visitation using the same instrument with the record set at 25 mm/s and a voltage calibration of 1mV/cm. The ECGs were analyzed by a trained cardiologist, who was unaware of the patients’ clinical and paraclinical information. BBBs and fascicular blocks were reported based on universal definitions. If the patients did not fulfill the definition of any of the typical blocks, they were considered to be intraventricular conduction delays. The fQRS was defined as a narrow QRS complex (<120 ms) with a notch between the Q wave and the S wave in at least 2 related leads; BBBs were excluded. Given the association between ischemic heart disease and the fQRS, coronary heart disease was considered to be a confounding factor for this study.^12^^, ^^13^ Its confounding effect was eliminated by employing the Framingham risk score as an indirect, noninvasive, and predictive marker for coronary heart disease.^   14 ^ The Framingham risk score was also calculated.^   15 ^

The continuous variables were checked for normal distribution with the Shapiro–Wilk test. Except for the variable of age, all the others did not have a normal distribution. Consequently, the χ^2^ test, the Mann–Whitney U test, and the Spearman correlation test were applied. The P values were adjusted via the Benjamini–Hochberg method for the false discovery rate, and values lower than 0.05 were considered significant. All the statistical analyses were performed with the SPSS software for Windows version 19.0 (Armonk, NY: IBM Corp.).

## Results

In the present study, 41 patients with SSc were enrolled (82.9% female and 17.1% male). The mean age of the studied population was 47.48±11.57 years old. The median disease duration was 7.00 years, with an interquartile range (IQR_25–75%_) of 4.00 to 11.75 years and the maximum and minimum of scores of 1 and 26 years, respectively. Among these individuals, 43.9% had diffuse cutaneous SSc, and the remainder had limited cutaneous SSc. Hypertension, smoking, and diabetes mellitus were also reported in 9, 4, and 8 cases, respectively. The median Framingham risk score was 3.9 (IQR_25–75%_: 1.7–7.9), with a minimum score of 1 and a maximum score of 30. The median skin MSSS was 2 (IQR_25–75%_: 1–2), with a minimum score of 1 and a maximum score of 4. The median lung MSSS score was 1 (IQR_25–75%_: 0–2) in the range of 0 to 3. Moreover, the median vascular MSSS was 1 (IQR_25–75%_: 1–2), with the minimum and maximum scores of 0 and 4, respectively. 

Complete and incomplete BBBs were totally present in 26.8% of the patients. Left anterior hemiblocks (LAHBs) were the most prevalent abnormality (5 [12.1%] patients), followed by right bundle branch blocks (RBBBs) in 4 (9.7%) patients. Incomplete RBBBs, left posterior hemiblocks, and intraventricular conduction delays were identified in 4.8%, 2.4%, and 2.4%, respectively. The fQRS was found in 36.5% of the entire patients; this finding was more prevalent in the inferior leads: 11 out of 15 cases had this phenomenon in II, III, and aVF leads. Afterward, 3 cases were detected in the high lateral leads (I and aVL), 1 case in the anteroseptal leads (V_1 _and V_2_), and 1 case in the anterior precordial leads (V_3_–V_6_). The association between the fQRS and the clinicodemographic indices is shown in Table 1, and the association between the indicated variables and the BBB is depicted in Table 2. 

**Table 1:** Association between the fQRS and the demographic and clinical variables*

	fQRS (N=41)			
	Present (n=15)	Absent (n=26)	P	OR (95%CI)
SSc subtype			0.028	0.10 (0.02 to 0.56)
lcSSc	12 (85.7)	9 (37.5)		
dcSSc	2 (14.3)	15 (62.5)		
Disease duration (y)	6.0 (4.0-13.0)	8.0 (4.0-11.5)	0.705	0.51 (-3.27 to 4.28)
Gender			0.705	0.72 (0.14 to 3.80)
Male	3 (20.0)	4 (15.4)		
Female	12 (80.0)	22 (86.4)		
Framingham risk score	5.3 (1.7-13.7)	3.05 (1.6-7.3)	0.638	2.24 (-2.32 to 6.81)
MSS-skin	1.0 (1.0-2.0)	2.0 (1.0-2.0)	0.259	0.33 (-0.07 to 0.73)
MSS-lung	0.0 (0.0-1.0)	1.0 (0.0-2.0)	0.339	0.46 (-0.25 to 1.16)
MSS-vascular	1.0 (1.0-1.0)	1.0 (1.0-2.0)	0.273	0.43 (-0.08 to 0.94)

fQRS, Fragmented QRS; SSc, Systemic sclerosis; lcSSc, Limited cutaneous SSc; dcSSc, Diffuse cutaneous SSc; MSS, Medsger severity score

* Data are presented as n (%) or median (IQR_25–75%_).

**Table 2:** Association between either complete or incomplete BBBs and the demographic and clinical variables*

	BBB			
	Present (n=11)	Absent (n=30)	P	OR (95%CI)
SSc subtype				
lcSSc	4 (40.0)	17 (60.7)	0.512	2.32 (0.53 to 10.13)
dcSSc	6 (60.0)	11 (39.3)		
Disease duration (y)	6.0 (3.5-17.3)	8.5 (4.0-11.3)	0.790	1.00 (-3.21 to 5.21)
Gender				
Male	3 (27.3)	4 (13.3)	0.512	0.41 (0.08 to 2.23)
Female	8 (72.7)	26 (86.7)		
Framingham risk score	2.8 (1.6-4.5)	5.6 (1.7-13.4)	0.512	4.62 (-0.18 to 9.41)
MSS-skin	2.0 (1.0-2.0)	1.5 (1.0-2.0)	0.790	0.04 (-0.42 to 0.49)
MSS-lung	0.0 (0.0-2.0)	1.0 (0.0-2.0)	0.790	0.03 (-0.75 to 0.82)
MSS-vascular	1.0 (1.0-2.0)	1.0 (1.0-1.25)	0.512	0.40 (-0.16 to 0.96)

BBB, Bundle branch block; SSc, Systemic sclerosis; lcSSc, Limited cutaneous SSc; dcSSc, Diffuse cutaneous SSc; MSS, Medsger severity score

* Data are presented as n (%) or median (IQR_25–75%_).

## Discussion

In the present study on an Iranian SSc population, the prevalence rates of BBBs and the fQRS were 26.8% and 36.5%, respectively. Moreover, no associations were identified between these ECG findings and cutaneous, pulmonary, or vascular involvement. Described previously, the MSSS was used as an accepted tool to semi-quantify the severity of organ involvement in SSc.^   10 ^ The conduction abnormalities are also more prevalent in non–SSc-related cardiovascular diseases. Accordingly, the Framingham risk score was used in the current study to measure the likelihood of developing cardiovascular diseases due to conventional non–SSc-related risk factors.^   16 ^ The mean Framingham risk score was not statistically different between the individuals with and without conduction abnormalities. This finding may indicate that the non–SSc-related risk factors did not interpret the results of this study. 

In the current work, the fQRS was found in 36.5% of the patients, of which 73.3% was present in the inferior leads (III and aVF). The fQRS was detected in 57.1% of the patients with limited cutaneous SSc, compared with 11.7% in the patients with diffuse cutaneous SSc with the adjusted P value of 0.028 and the OR (95% CI) of 0.100 (0.018-0.553). This finding is not compatible with most of the previously reported studies indicating the higher prevalence of cardiac involvement in patients with diffuse cutaneous SSc.^   17 ^ The other clinical or demographic variables, including the lung MSSS consisting of the estimated PAP, were not associated with the fQRS. In an investigation performed on approximately 11000 middle-aged healthy subjects, the fQRS was detected in 18% of the individuals with a predominance in the inferior leads.^        18 ^ According to this prevalence in the normal population, it appears that the fQRS is more prevalent among patients with SSc. In line with our findings, Tigen et al.^        19 ^ revealed a 24.5% prevalence of the fQRS among their patients with SSc, with 84% of the cases seen in the inferior leads and without any correlation with the echocardiographic findings. Moreover, Bayar and colleagues^   20 ^ found the fQRS in 54% of their studied patients with SSc and reported an association with the PAP measured similarly by echocardiography. The predominance of the fQRS in the inferior leads reported currently and in the mentioned studies can be justified by the higher prevalence of cardiac fibrosis in the inferior parts of the heart studies by cardiac magnetic resonance imaging in patients with SSc.^        21 ^ Thus far, there has been no evidence regarding the prognostic significance of the fQRS in SSc; accordingly, it is necessary that this matter be promptly investigated in the future. 

In line with the probable linkage of the extracellular matrix deposit and the fQRS, the presence of this ECG finding is reported in the other types of cardiomyopathies. Amyloidosis is a disease in which different proteins from different sources are deposited in variable organs and even in the myocardium. In one study, the fQRS was detected in 23.5% of patients with amyloid light-chain (AL) amyloidosis, with a significantly higher percentage in individuals with cardiac involvement.^        22 ^ Sixty percent of the fQRS was reported in the inferior leads (II, III, and aVF), which is compatible with the same findings in SSc. Another disease with a partially similar etiology is sarcoidosis, in which the granuloma formation can occur in any organ, including the myocardium. According to a study by Homsi et al.,^        23 ^ the fQRS was found in 46% of the pulmonary type and 75% of cardiac sarcoidosis. In addition to the relation between the fQRS and extra-cellular abnormal deposition, the inflammatory origin of this ECG finding has also been previously proposed. Sayin and colleagues^        24 ^ reported a 54% prevalence of the fQRS in 43 patients with Behçet’s disease, which was associated with a prolonged disease duration and elevated C-reactive protein levels. The autoimmune nature of Behçet’s disease and the correlation with acute phase reactants, thus, suggest the possibility of the inflammatory nature of the fQRS. 

The prevalence of either complete or incomplete BBBs was 26.8% in this study. The most prevalent conduction block was the LAHB, which was present in 12.1% of the patients. According to a study conducted on 8000 cases by Yano et al.,^   25 ^ LAHBs were found in approximately 2.5% of the normal population. Another study on 3978 healthy individuals showed a prevalence of 0.3% for this conduction abnormality.^   26 ^ Consequently, it appears that LAHBs are more prevalent in patients suffering from SSc than in the normal population. Similar to our findings, Pourmoghim and colleagues^   27 ^ also reported that LAHBs were the most common conduction abnormality (8.8%) in a sample of Iranian patients with SSc. Furthermore, in a study by Follansbee et al.,^28^ LAHBs were also the most prevalent BBBs (10% of the cases with SSc). Similarly, in a study by Roberts et al.,^28^^, ^^29^ LAHBs were present in 16% of the cases. 

We found complete and incomplete RBBBs in 9.7% and 4.8% of our patients, correspondingly. According to the Copenhagen City Heart Study, which was carried out on 18441 healthy cases, the rates of RBBBs/incomplete RBBBs were reported to be 1.4%/4.7% in men and 0.5%/2.3% in women.^   30 ^ Consequently, it appears that this conduction abnormality is also more prevalent in patients suffering from SSc. According to a study by Draeger et al.,^   31 ^ RBBBs/incomplete RBBBs were reported in 2.6%/0.75% and RBBBs were associated with a higher risk of mortality (HR: 5.3 [95%CI: 2.1-13.4] and P<0.001). Comparable to our results, RBBBs were not associated with the PAP in the mentioned study. In the present work, we detected no left bundle branch blocks (LBBBs) in any of our patients. The prevalence of LBBBs was reported to be between 0.1% and 0.8% in the normal population^   26 ^ and between 2.6% and 3% in patients with SSc. It is probable that the different patient populations were the cause of these varieties, and the cause of this difference needs to be further studied. 

The present study has some limitations that should be explained. As we have discussed previously, the conduction abnormalities in the current work were associated with non–SSc-related conventional risk factors and cardiovascular diseases, the most notable of which are atherosclerosis and ischemic heart disease. Given the high cost and lack of the indication for most of the cases, we utilized the Framingham risk score instead of coronary angiography. Additionally, a lack of a control group and the low number of the patients are the other salient limitations of this study. 

## Conclusion

 It seems that BBBs and the fQRS are more prevalent in patients with SSc. Given the lack of an association between these conduction abnormalities and the Framingham risk score, it is likely that non–SSc-related cardiovascular diseases do not justify this high prevalence of conduction abnormalities in SSc. Moreover, the studied conduction blocks were not associated with cutaneous, pulmonary, and vascular involvements. It is likely that the pathogenesis of the cardiac involvement in SSc may be independent of the involvement of the other organs. 

## Notes:

***This paper should be cited as: ***Javinani A, Javady Nejad Z, Gharibdoost F, Jamshidi A. R, Atef Yekta R, Alvand S, Imeni V, Hashemi SN, Kavosi H. Bundle Branch Blocks and Fragmented QRS Complex in Iranian Patients with Systemic Sclerosis. J Teh Univ Heart Ctr 2019;14(1):6-11.
